# Comprehensive Evaluation of Anterior Corneal Change in Asphericity Calculated by the Tangential Radius of Curvature after LASIK

**DOI:** 10.1155/2017/3874371

**Published:** 2017-02-06

**Authors:** Jinglu Ying, Jianqiu Cai, Leru Zhu, Yi Zha

**Affiliations:** ^1^Department of Ophthalmology, Sir Run Run Shaw Hospital, Zhejiang University School of Medicine, 3 East Qingchun Road, Hangzhou 310016, China; ^2^Department of Ophthalmology, The Second Affiliated Hospital and Yuying Children's Hospital of Wenzhou Medical University, No. 109, West-Xueyuan Road, Wenzhou 325027, China; ^3^Department of Ophthalmology, The First Affiliated Hospital of Wenzhou Medical University, No. 2, Fuxue Lane, Wenzhou 325000, China

## Abstract

To evaluate the change in the anterior corneal asphericity (Δ*Q*) comprehensively calculated using the tangential radius (*r*_*t*_) after LASIK. Forty-two right eyes were evaluated using the Orbscan II corneal topographer. The pre- and postoperative *Q*-values of the flat principal semimeridians calculated by the sagittal radius were compared to those by the tangential radius. The *Q*-value of each semimeridian in the horizontal region was calculated by *r*_*t*_. Fourier fitting was used to model the 360-semimeridional variation of *Q*-values and to fit the *Q*-values in the vertical region before and after surgery. There were significant differences in *Q*-values between the two methods before (*P* < 0.001) and after surgery (*P* = 0.003). A significant increase in postoperative *Q*-value was detected compared to preoperative *Q*-value (*P* < 0.001) calculated by *r*_*t*_. The 360-semimeridional variation of the *Q*-values was well fitted with a third- and fourth-degree Fourier function before and after surgery. The Δ*Q*-value distribution presented double valley variation, with the amount of Δ*Q* being lowest in the near-vertical regions and highest in the near-horizontal regions. Calculating the *Q*-value with *r*_*t*_ combined with Fourier fitting, we evaluated 360 Δ*Q*-values' variation of semimeridians of the entire anterior corneal surface and then displayed true and complete anterior corneal shape after LASIK.

## 1. Introduction

Laser in situ keratomileusis (LASIK) is the most frequently performed corneal refractive surgery for myopia and astigmatism [1]. Asphericity can be defined to variations in radius of curvature from apex to periphery and mathematically described to be a *Q*-value [2]. Most previous studies reported that LASIK induces a positive change in the anterior corneal asphericity (Δ*Q*) after myopic ablation [3–5].

The reported *Q*-values obtained by corneal topographers in previous studies were calculated using the sagittal radius of curvature (*r*_*s*_) according to Bennett's equation [6]. The corneal topographers can only provide *Q*-values of two principal corneal meridians or four principal corneal semimeridians. Our previously published papers [7–9] had found that the *Q*-value calculated by *r*_*s*_ is significantly different from that by tangential radius of curvature (*r*_*t*_) for normal emmetropic eyes and demonstrated that *Q*-value calculation by *r*_*t*_ can provide more accurate *Q*-values than that by *r*_*s*_. In addition, the method of calculating corneal *Q* using *r*_*t*_ can obtain *Q*-values of any semimeridian and we had showed the 360-semimeridional variation of *Q*-values calculated by *r*_*t*_ for normal emmetropic eyes.

The purposes of present study are to (1) compare *Q*-values in the flat principal semimeridians between that calculated by the sagittal radius (*r*_*s*_) and that by the tangential radius (*r*_*t*_) before and after LASIK; (2) compare pre- and postoperative *Q*-value calculated by *r*_*t*_; (3) evaluate 360 Δ*Q*-values' variation of semimeridians of the entire anterior corneal surface calculated by the tangential radius (*r*_*t*_) and displayed true and complete anterior corneal shape after LASIK for the first time.

## 2. Materials and Methods

The study was designed as a retrospective, consecutive study. Informed consent was obtained from all patients. The study was approved by the institutional review board and adhered to the tenets of the Declaration of Helsinki.

Preoperative evaluation included the corrected distance visual acuity, uncorrected distance visual acuity, manifest refraction, slit-lamp biomicroscopy, fundus examination, and corneal topography. All examinations were performed at 1, 3, and 6 months postoperatively. Patients with active systemic or ocular disease, previous ocular surgery and ocular trauma were excluded from the study.

Corneal topography of the right eye for each subject was done using scanning-slit technology with the Bausch & Lomb Orbscan II corneal topographer (version 3.00). Three images were obtained from each subject. The sagittal radius of curvature (*r*_*s*_), the tangential radius of curvature (*r*_*t*_), perpendicular distance from the point to the optical axis (*y*) of all data points on a semimeridian, and vertex radius of curvature (*r*_0_) were obtained from the axial and tangential power map of the anterior corneal surface. The data points were arranged on a semimeridian at 0.1-mm intervals. The interval between two semimeridians was 1 deg. Anterior corneal astigmatism (3 and 5 mm) was also recorded.

### 2.1. Surgical Technique

LASIK was performed using the NIDEK EC-5000 II scanning excimer laser platform with conventional ablation. A superiorly hinged corneal flap was created using the Moria2 microkeratome. The optical zone ranged from 6.0 to 6.5 mm, and the transition zone extended to 6.5 to 7.0 mm. The same surgeon (CJQ) performed all operations. In all cases, the targeted postoperative refraction was emmetropia.

### 2.2. *Q*-Value Calculation by the Sagittal Radius of Curvature

*Q*-value of semimeridian was calculated by *r*_*s*_ using Bennett's equation [6] as follows:

(1) $$\begin{array}{c} {r_{s}}^{2}={r_{0}}^{2}+\left(\;-Q\;\right)y^{2}. \end{array}$$

A scatterplot of *r*_*s*_^2^ (on the ordinate) against *y*^2^ (on the abscissa) can produce a straight line function. The negative slope of the line equals *Q*. The straight line gives a coefficient of determination (*R*^2^). The *Q*-value of the flat principal semimeridian was calculated by the points from the first point at 0.1 mm to the peripheral point at 3.5 mm on the axial power map. The mean of three *Q*-values of the flat principal semimeridian was considered the final value.

### 2.3. *Q*-Value Calculation by the Tangential Radius of Curvature

Our previously published papers [7–9] have introduced the derivation of the equation in detail for the *Q*-value calculation according to the tangential radius. The equation could be expressed as

(2) $$\begin{array}{c} r_{t}=\frac{1}{{r_{0}}^{2}}{\left[\;{r_{0}}^{2}-Qy^{2}\;\right]}^{3/2}. \end{array}$$

Equation (2) was converted into the form *y*^2^ = *b* + *cr*_*t*_^2/3^, where *b* and *c* are constants. A scatterplot of *y*^2^ (on the ordinate) against *r*_*t*_^2/3^ (on the abscissa) can produce a straight line function. Using linear regression, we obtain *Q* = −*b*^2^/*c*^3^. The straight line gives the coefficient of determination (*R*^2^). The *Q*-value of a given semimeridian was calculated by the points from the first point at 0.1 mm to the peripheral point at 3.5 mm on the tangential power map. The mean of three *Q*-values of a given semimeridian was considered the final value.

### 2.4. Modeling the 360-Semimeridional Variation Rule of the *Q*-Value by Tangential Radius

We previously found that the horizontal region showed a good coefficient of determination (*R*^2^), whereas the coefficient of determination for the vertical region was relatively poor. Thus, according to the *Q*-value of each semimeridian in the horizontal regions, including 0–50°, 130–180°, 181–230°, and 310–359°, the 360-semimeridional variation of the *Q*-values for each subject was modeled using Fourier fitting with MATLAB (MathWorks, Inc.). Then, we fit the *Q*-value of each semimeridian in the vertical regions, including 51–129° and 231–309°. The Fourier function took the following form:

(3) fx=a0+a1∗cos⁡x∗w+b1∗sin⁡x∗w+a2∗cos⁡2∗x∗w+b2∗sin⁡2∗x∗w+⋯,

where *x* is the semimeridian angle *θ* (degree) and *f*(*x*) is the corresponding *Q*-value. The degree was converted to a radian when we performed Fourier fitting. The Fourier fitting gave the goodness of fit (*r*^2^) and root mean square error (RMSE).

### 2.5. Statistical Analysis

Statistical analysis was performed using SPSS software (version 17.0, SPSS, Inc.). The Kolmogorov-Smirnov test was used to evaluate whether the data had a normal distribution. The level of significance was set at five percent. Considering the reliability of the linear regression equation in the *Q*-value calculation, the coefficient of determination (*R*^2^) should be more than 0.5. Differences between *Q*-values of the flat principal semimeridians by the two methods were compared by paired *t*-test. Comparison of preoperative and postoperative *Q*-value calculated by *r*_*t*_ was analyzed by paired *t*-test. Univariable regression analysis was performed with the preoperative spherical equivalent (SE) and preoperative anterior corneal astigmatism considered as independent variables and Δ*Q* as a dependent variable. The Pearson correlation coefficient (*r*) was used to assess the correlation between different variables.

## 3. Results

Forty-two right eyes from 42 patients (15 females and 27 males) were evaluated at 3 to 6 months. The mean subject age was 24 ± 5.46 years (SD) (range: 17 to 38 years). All eyes had myopia with or without astigmatism and a mean spherical equivalent refractive error (SE) of −6.00 ± 2.22 D (range: −3.00 D to −11.25 D). No intraoperative or postoperative complications were detected.

### 3.1. Function Relationship

**Figure 1:**
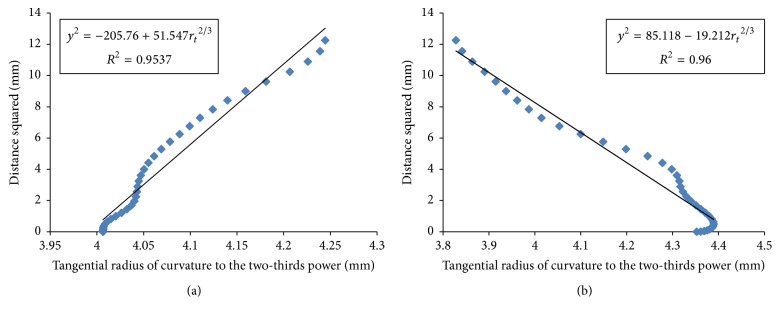
Scatterplot of distance squared (*y*^2^) versus tangential radius of curvature to the two-thirds power (*r*_*t*_^2/3^) in the nasal flat principal semimeridian of the right eye for subject number one before (a) and after surgery (b).

**Figure 2:**
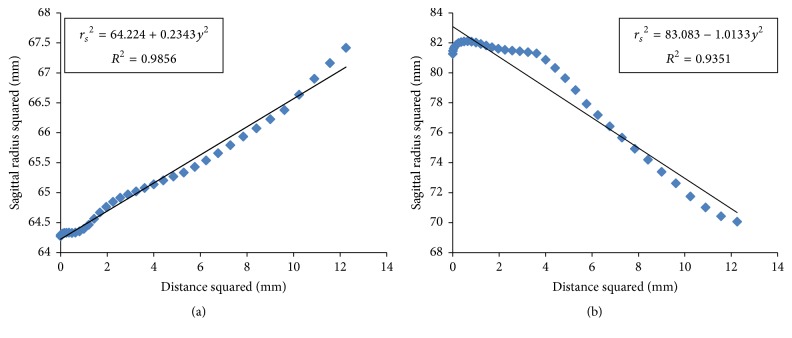
Scatterplot of sagittal radius squared (*r*_*s*_^2^) versus distance squared (*y*^2^) in the nasal flat principal semimeridian of the right eye for the same subject before (a) and after surgery (b).

Figure 1 shows the function scatterplot of distance squared (*y*^2^) versus the tangential radius of curvature to the two-thirds power (*r*_*t*_^2/3^) in the nasal flat principal semimeridian of the right eye for subject number one before and after surgery.  Figure 2 shows the function scatterplot of sagittal radius squared (*r*_*s*_^2^) versus distance squared (*y*^2^) in the nasal flat principal semimeridian of the right eye of the same subject before and after surgery.

### 3.2. Comparison of *Q*-Values between the Two Methods in the Flat Principal Semimeridians before and after Surgery

Table 1 shows the mean pre- and postoperative *Q*-values in the flat principal semimeridians calculated by sagittal and tangential radius. There were significant differences in *Q*-values between the two methods before (*P* < 0.001) and after surgery (*P* = 0.003). We found that the preoperative *Q*-values were more negative calculated by the tangential radius than those by the sagittal radius; the postoperative *Q*-values were more positive calculated by the tangential radius than those by the sagittal radius.

### 3.3. 360-Semimeridional Variation Rule of the *Q*-Value by Tangential Radius

To determine what degree of Fourier function would provide an optimal fit to the 360-semimeridional variation of the *Q*-value, we calculated the RMSE of the fit of the Fourier function from the third to sixth degrees. For preoperation, the RMSE was relatively stable at approximately 0.02 for fits higher than the second degree. The 360-semimeridional variation of the *Q*-value was well fitted with a third-degree Fourier function for all subjects. The mean value of goodness of fit (*r*^2^) was 0.94 ± 0.03. The mean RMSE value was 0.02 ± 0.007. For postoperation, the RMSE was relatively stable at approximately 0.05 for fits higher than the third degree. The 360-semimeridional variation of the *Q*-value was well fitted with a fourth-degree Fourier function for all subjects. The mean value of goodness of fit (*r*^2^) was 0.95 ± 0.02. The mean RMSE value was 0.05 ± 0.02.

**Figure 3:**
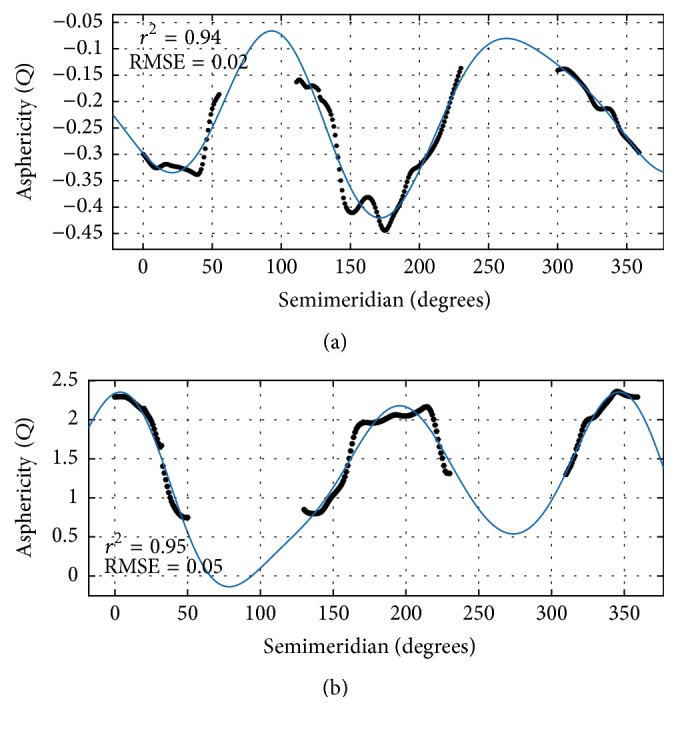
Typical example of the asphericity variation (*Q*) as a function of semimeridian for subject number 15 before (a) and after (b) surgery. Black: the *Q*-value of each semimeridian in the horizontal region. Blue: the fitted curve of the *Q*-value 360-semimeridional variation. *r*^2^: goodness of fit of the Fourier function. RMSE: root mean square error.

Figure 3 shows an example of the variation of the asphericity (*Q*) as a function of the semimeridian for subject number 15 before and after surgery. The corresponding preoperative and postoperative Fourier functions are as follows:

(4) fxpre=−0.22+0.03cos⁡0.017x−0.03sin⁡0.017x−0.13cos⁡0.034x−0.008sin⁡0.034x+0.025cos⁡0.051x−0.04sin⁡0.051x,fxpost=1.145+0.019cos⁡0.018x−0.44sin⁡0.018x+0.88cos⁡0.036x+0.319sin⁡0.036x+0.2584cos⁡0.054x−0.037sin⁡0.054x+0.035cos⁡0.072x+0.093sin⁡0.072x.

**Figure 4:**
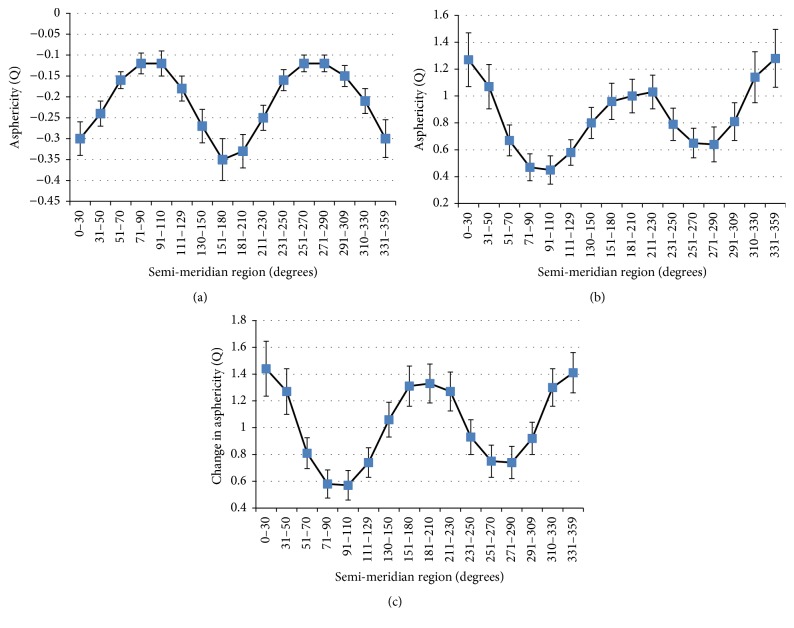
Variation in the asphericity (*Q*) as a function of the semimeridian region of the anterior corneal surface for all subjects. Bars denote the 95% confidence interval (CI). (a) Preoperation; (b) postoperation; (c) the pre- and postoperative change in *Q* (Δ*Q*).

The preoperative *Q*-values for the sample analyzed in our study had negative values (mean: −0.22 ± 0.01), which gradually became less negative from the horizontal to vertical semimeridian regions in each quadrant. The *Q*-value distribution of the anterior corneal surface had a bimodal variation; the two peak values represented the least-negative *Q*-values (Figure 4(a)). Postoperatively, the *Q*-values in our study had positive values (mean: +0.82 ± 0.32), which gradually became less positive from the horizontal to vertical semimeridian regions in each quadrant. The *Q*-value distribution of the anterior corneal surface presented with a double valley variation; the two valley values represent the least-positive *Q*-values (Figure 4(b)). A significant increase in the mean postoperative *Q*-value was detected compared to the mean preoperative *Q*-value (*P* < 0.001).  Table 2 shows the mean values of Δ*Q* at different semimeridian regions in the four quadrants of the anterior corneal surface. The mean Δ*Q* was +1.05 ± 0.32. The distribution of Δ*Q* also presented with a double valley variation; +Δ*Q* was lowest in the near-vertical regions (90–110° and 251–270°) and highest in the near-horizontal regions (0–30° and 331–359°) (Figure 4(c)).

Δ*Q* was highly correlated with the preoperative SE (*P* = 0.000, *r* = 0.805) and mildly correlated with the preoperative anterior corneal astigmatism (5 mm) (*P* = 0.017, *r* = 0.366). The higher the preoperative SE, the more positive the postoperative *Q*-value. For each diopter of myopic treatment, there was a +0.14 increase in the *Q*-value. The greater the preoperative anterior corneal astigmatism was, the more positive the postoperative *Q*-value. For each per diopter increase in the anterior corneal astigmatism, there was a +0.2 increase in the *Q*-value.

## 4. Discussion

In our present study, we found that the *Q*-values calculated by the tangential radius were significantly different from those by the sagittal radius before and after LASIK. This result was in agreement with the finding of our previous paper, which have evaluated normal emmetropic eyes [7]. The tangential radius of curvature (*r*_*t*_) is a true radius of curvature that better represents the corneal shape and local curvature changes [10]. However, as the sagittal radius of curvature is spherically biased, it is not a true radius of curvature [11–13]. In addition, we had demonstrated that *Q*-value calculation by *r*_*t*_ can provide more accurate and complete *Q*-values than that by *r*_*s*_ in previous study [7]. Thus, we chose tangential radius to calculate the anterior corneal asphericity before and after LASIK as well as to evaluate 360 Δ*Q*-values' variation of semimeridians of the entire anterior corneal surface and displayed true and complete anterior corneal shape after LASIK for the first time.

The present study found that LASIK induced a positive change in the anterior corneal asphericity. The mean *Q*-value after LASIK was +0.82 ± 0.32, and the mean +Δ*Q* was +1.05 ± 0.32 in the direction of a more oblate profile. Kamiya et al. [14] found that the mean *Q*-value was +0.42 ± 0.30 after FLEX and +0.65 ± 0.30 after wavefront-guided LASIK using a microkeratome. Bottos et al. [15] reported that the mean +Δ*Q* was +0.63 ± 0.44 after wavefront-guided LASIK using a femtosecond flap. Molchan et al. [16] found that the mean +Δ*Q* was +0.39 ± 0.20 after wavefront-optimized LASIK and +0.54 ± 0.26 after wavefront-guided LASIK using PRK. El Danasoury et al. [17] found that the mean *Q*-value was +0.07 ± 0.26 after LASIK with an optimized prolate ablation (OPA) and +0.30 ± 0.26 after LASIK with conventional ablation using a femtosecond flap. Goyal et al. [18] found that the mean +Δ*Q* was +0.53 ± 0.31 after wavefront-optimized LASIK and +0.91 ± 0.30 after wavefront-guided LASIK using a microkeratome. Our results were much greater than those of the above studies. The likely causes for this difference are as follows. (1) We calculated the corneal *Q*-value by the tangential radius instead of the sagittal radius. The postoperative *Q*-values were more positive calculated by the tangential radius than those by the sagittal radius. (2) The mean preoperative SE of our study was −6.00 D greater than that of the above studies, ranging from −4.00 to −5.00 D. (3) Δ*Q* induced by LASIK with conventional ablation was higher than LASIK with customized ablation. (4) We analyzed the corneal *Q*-value for the 7 mm corneal diameter, which was representative of all semimeridians, while other studies analyzed the *Q*-value for 6 mm, which is representative of two principal meridians or four principal semimeridians. In addition, Choi et al. [19] reported that the mean +Δ*Q* was +1.04 after LASIK for optical path difference customized aspheric treatment and +0.58 after LASIK for OPA using PRK in high myopic eyes. Vega-Estrada et al. [20] found that the mean *Q*-value was +0.61 ± 0.63 for a 4.5 mm diameter and +1.15 ± 0.45 for an 8.0 mm diameter in highly myopic eyes after LASIK with an optimized ablation profile using a femtosecond flap.

The 360-semimeridional variation of the *Q*-value was well fitted with the Fourier function for all subjects before and after surgery. The 360-semimeridional variation of *Q*-values in individuals in Figure 3 was similar to that in the subjects in Figures 4(a) and 4(b). Preoperatively, the *Q*-value distribution of the anterior corneal surface had bimodal variation; postoperatively, it had double valley variation. These represent the least-negative and least-positive *Q*-values, respectively. The distribution of Δ*Q* presented double valley variation; +Δ*Q* was lowest in the near-vertical regions (+0.55 in 90–110°; +0.75 in 251–270°) and highest in the near-horizontal regions (+1.54 in 0–30°; +1.56 in 331–359°).

Also, we found that Δ*Q* was positively correlated with the preoperative SE (*r* = 0.805). For each diopter of myopic treatment, there was a +0.14 increase in the *Q*-value. Bottos et al. [15] reported that Δ*Q* per diopter of treatment SE was +0.18 after wavefront-guided LASIK. Molchan et al. [16] found that Δ*Q* per diopter of treatment SE was +0.12 after wavefront-optimized LASIK and +0.14 after wavefront-guided LASIK. Vega-Estrada et al. [20] reported that, for each diopter of spherical correction, asphericity (8 mm) increased by 0.223 postoperatively in high myopic eyes after LASIK performed with an optimized ablation profile. In addition, they found that the postoperative *Q* (4.5 mm) was negatively correlated with the central corneal pachymetry and optical zone, and postoperative *Q* (8 mm) was negatively correlated with preoperative sphere and ablation zone. Furthermore, we found that Δ*Q* was mildly correlated with the preoperative anterior corneal astigmatism (5 mm) (*r* = 0.366). For each per diopter increase in the anterior corneal astigmatism, there was a +0.2 increase in the *Q*-value.

In conclusion, our study found that there were significant differences in *Q*-values between the sagittal radius and the tangential radius before and after LASIK. We also found that myopic LASIK induced a positive change in the anterior corneal asphericity calculated by the tangential radius of curvature (*r*_*t*_). In addition, we evaluated the 360 Δ*Q*-values' variation of semimeridians of the entire anterior corneal surface and displayed the true and complete anterior corneal shape after LASIK. Furthermore, we found that the greater the preoperative SE and anterior corneal astigmatism (5 mm) was, the greater the induction of anterior corneal asphericity after LASIK. Further work is needed to compare Δ*Q* that is calculated by the tangential radius of curvature after small incision lenticule extraction (SMILE) and Femtosecond Laser-Assisted LASIK (FS-LASIK). The model of the whole anterior corneal surface would be reconstructed after SMILE and FS-LASIK surgery.

## Figures and Tables

**Table 1:** Pre- and Postoperative values for asphericity (*Q*) in the flat principal semimeridians calculated by sagittal and tangential radius of curvature for the right eyes.

		Sagittal	Tangential	*N*	*P*
Preoperative					
*Q*	Nf	−0.26 ± 0.16	−0.34 ± 0.15	42	<0.001
*Q*	Tf	−0.28 ± 0.16	−0.37 ± 0.17	42	<0.001
Postoperative					
*Q*	Nf	0.80 ± 0.38	0.97 ± 0.34	42	0.003
*Q*	Tf	0.82 ± 0.46	0.99 ± 0.45	42	0.003

Nf: nasal flat principal semimeridian; Tf: temporal flat principal semimeridian. *n* = number of eyes.

**Table 2:** Values for the change in the asphericity (ΔQ) at different semimeridian regions in four quadrants of the anterior corneal surface.

Corneal semimeridian region (degrees)																
	0–30	31–50	51–70	71–90	91–110	111–129	130–150	151–180	181–210	211–230	231–250	251–270	271–290	291–309	310–330	331–359
Mean ± SD	1.44 ± 0.46	1.27 ± 0.47	0.81 ± 0.34	0.58 ± 0.32	0.57 ± 0.34	0.74 ± 0.32	1.06 ± 0.40	1.31 ± 0.44	1.33 ± 0.45	1.27 ± 0.45	0.93 ± 0.40	0.75 ± 0.35	0.74 ± 0.39	0.92 ± 0.43	1.30 ± 0.52	1.41 ± 0.45
Range	0.68 to 2.54	0.55 to 2.30	0.17 to 1.53	0.05 to 1.28	0.05 to 1.57	0.23 to 1.47	0.42 to 2.11	0.53 to 2.32	0.53 to 2.40	0.51 to 2.26	0.35 to 1.86	0.11 to 1.56	0.11 to 1.56	0.17 to 2.17	0.45 to 2.52	0.73 to 2.54

*n* = number of eyes; SD = standard deviation.

## References

[1] Sandoval H. P., de Castro L. E. F., Vroman D. T., Solomon K. D. (2005). Refractive surgery survey 2004. *Journal of Cataract and Refractive Surgery*.

[2] Gatinel D., Haouat M., Hoang-Xuan T. (2002). A review of mathematical descriptors of corneal asphericity. *Journal Francais d'Ophtalmologie*.

[3] Hersh P. S., Fry K., Blaker J. W. (2003). Spherical aberration after laser in situ keratomileusis and photorefractive keratectomy: clinical results and theoretical models of etiology. *Journal of Cataract and Refractive Surgery*.

[4] Holladay J. T., Dudeja D. R., Chang J. (1999). Functional vision and corneal changes after laser in situ keratomileusis determined by contrast sensitivity, glare testing, and corneal topography. *Journal of Cataract and Refractive Surgery*.

[5] Holladay J. T., Janes J. A. (2002). Topographic changes in corneal asphericity and effective optical zone after laser in situ keratomileusis. *Journal of Cataract and Refractive Surgery*.

[6] Bennett A. G., Rabbetts R. B. (1991). What radius does the conventional keratometer measure?. *Ophthalmic and Physiological Optics*.

[7] Ying J., Wang B., Shi M. (2012). Anterior corneal asphericity calculated by the tangential radius of curvature. *Journal of Biomedical Optics*.

[8] Zheng S., Ying J., Wang B., Xie Z., Huang X., Shi M. (2013). Three-dimensional model for human anterior corneal surface. *Journal of Biomedical Optics*.

[9] Wang B., Huang X., Ying J., Shi M. (2013). The 3D computer image of the anterior corneal surface. *Engineering*.

[10] Szczotka L. B., Thomas J. (1998). Comparison of axial and instantaneous videokeratographic data in keratoconus and utility in contact lens curvature prediction. *CLAO Journal*.

[11] Roberts C. (1994). Characterization of the inherent error in a spherically-biased corneal topography system in mapping a radially aspheric surface. *Journal of refractive and corneal surgery*.

[12] Roberts C. (1995). Analysis of the inherent error of the TMS-1 topographic modeling system in mapping a radially aspheric surface. *Cornea*.

[13] Chan J. S., Mandell R. B., Burger D. S., Fusaro R. E. (1995). Accuracy of videokeratography for instantaneous radius in keratoconus. *Optometry and Vision Science*.

[14] Kamiya K., Shimizu K., Igarashi A., Kobashi H., Komatsu M. (2013). Comparison of visual acuity, higher-order aberrations and corneal asphericity after refractive lenticule extraction and wavefront-guided laser-assisted in situ keratomileusis for myopia. *British Journal of Ophthalmology*.

[15] Bottos K. M., Leite M. T., Aventura-Isidro M. (2011). Corneal asphericity and spherical aberration after refractive surgery. *Journal of Cataract and Refractive Surgery*.

[16] Molchan R. P., Taylor K. R., Panday V. A., Caldwell M. C., Reilly C. D. (2015). Retrospective analysis comparing the preoperative and postoperative ‘Q’ values for 2 different lasers in refractive surgery. *Cornea*.

[17] El Danasoury A. M., Holladay J., Waring G. O., Pieger S., Bains H. S. (2012). A contralateral, randomized comparison of optimized prolate ablation and conventional LASIK for myopia with the NIDEK excimer laser platform. *Journal of Refractive Surgery*.

[18] Goyal J. L., Garg A., Arora R., Jain P., Goel Y. (2014). Comparative evaluation of higher-order aberrations and corneal asphericity between wavefront-guided and aspheric LASIK for myopia. *Journal of Refractive Surgery*.

[19] Choi B. J., Park Y. M., Lee J. S. (2015). Clinical outcomes between optical path difference custom aspheric treatment and optimized prolate ablation photorefractive keratectomy in myopia exceeding 8 diopters. *Eye (London)*.

[20] Vega-Estrada A., Alió J. L., Arba Mosquera S., Moreno L. J. (2012). Corneal higher order aberrations after LASIK for high myopia with a fast repetition rate excimer laser, optimized ablation profile, and femtosecond laser-assisted flap. *Journal of Refractive Surgery*.

